# A New Method to Predict Postoperative Stem Anteversion in Total Hip Arthroplasty for Developmental Dysplasia of the Hip

**DOI:** 10.1111/os.14037

**Published:** 2024-03-20

**Authors:** Yuehao Hu, Ziyang Sun, Jingwei Zhang, Mengning Yan, Yuanqing Mao, Huiwu Li, Degang Yu, Zanjing Zhai

**Affiliations:** ^1^ Shanghai Key Laboratory of Orthopaedic Implants, Department of Orthopaedic Surgery Shanghai Ninth People's Hospital, Shanghai Jiao Tong University School of Medicine Shanghai China; ^2^ Department of Orthopedics Shanghai Sixth People's Hospital Affiliated to Shanghai Jiao Tong University School of Medicine Shanghai China

**Keywords:** Developmental Dysplasia of the Hip, Femoral Anteversion, Stem Anteversion, Total Hip Arthroplasty

## Abstract

**Background:**

Preoperative evaluation of femoral anteversion to predict postoperative stem anteversion aids the selection of an appropriate prosthesis and optimizes the combined anteversion in total hip arthroplasty (THA) for developmental dysplasia of the hip (DDH). The conventional prediction methods are based on the femoral anteversion measurement at the location of the femoral head and/or neck. However, varied differences between femoral anteversion and postoperative stem anteversion were demonstrated. This study investigated the predictive role of a new method based on the principle of sagittal three‐point fixation.

**Methods:**

From January 2017 to December 2018, a total of 133 DDH hips that underwent THA were retrospectively analyzed. There were 76 Crowe type I, 27 type II, and 30 type III hips. The single‐wedge stem was used in 49 hips, and the double‐wedge stem was used in 84 hips. Preoperative native femoral anteversion at the femoral head–neck junction, anterior cortex anteversion at 2 levels of the lesser trochanter, posterior cortex anteversion at 5 levels of the femoral neck, and postoperative stem anteversion were measured using two‐dimensional computed tomography. Predictive anteversion by the new method was calculated as the average anteversion formed by the anterior cortex at the lesser trochanter and the posterior cortex at the femoral neck.

**Results:**

For hips with different neck heights, different Crowe types, different stem types, or different femoral anteversions, native femoral anteversion showed widely varied differences and correlations with stem anteversion, with differences ranging from −1.27 ± 8.33° to −13.67 ± 9.47° and correlations ranging from 0.122 (*p* = 0.705, no correlation) to 0.813. Predictive anteversion formed by the anterior cortex at the lesser trochanter proximal base and posterior cortex 10 mm above the lesser trochanter proximal base showed no significant difference with stem anteversion, with less varied differences (0.92 ± 7.52°) and good to excellent correlations (*r* = 0.826).

**Conclusion:**

Adopting our new method, predictive anteversion, measured as the average anteversion of the anterior cortex at the lesser trochanter proximal base and posterior cortex 10 mm above the lesser trochanter proximal base, predicted postoperative stem anteversion more reliably than native femoral anteversion.

## Graphical abstract

Our study provides a novel method to predictive postoperative stem anteversion based on the principle of sagittal three‐point fixation of the prosthesis, which measured as the average anteversion of the anterior cortex at the lesser trochanter proximal base and posterior cortex 10 mm above the lesser trochanter proximal base before the total hip arthroplasty surgery.

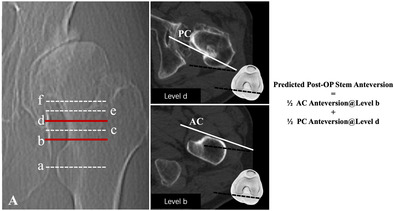

## Introduction

Total hip arthroplasty is an effective method to relieve pain and improve function for adult patients with developmental dysplasia of the hip, which is a disorder of abnormal development resulting in dysplasia, subluxation, and possible dislocation of the hip secondary to capsular laxity and mechanical instability.^1^ Abnormal femoral anteversion is a major anatomical abnormality in patients with DDH.^2^, ^3^ Preoperative estimation of femoral anteversion to predict postoperative stem anteversion and then intraoperatively adjusting the cup anteversion accordingly is one method often used in the “acetabular first” technique to target combined anteversion within the safe zone.^4^ At present, the cementless straight tapered femoral stem is the most commonly used stem. Its position depends mainly on the anatomy of the proximal femur, and it is difficult to adjust the stem anteversion intraoperatively.^5^ If the preoperative femoral anteversion angle is eccentric, a monoblock or modular stem with adjustable anteversion is preferable.^2^, ^3^ Therefore, preoperatively predicting the postoperative stem anteversion helps in the selection of an appropriate femoral prosthesis and optimizes the combined anteversion.

Computed tomography (CT) is thought to be the most accurate imaging method to assess femoral anteversion.^6^ Although several methods of femoral anteversion assessment have been reported to predict postoperative stem anteversion, no standard predictive method has been established.^7^, ^8^, ^9^, ^10^, ^11^ It remains controversial whether postoperative stem anteversion can be effectively predicted. Results of previous studies have varied widely,^7^, ^11^, ^12^, ^13^, ^14^ with the reported difference between preoperative femoral anteversion and postoperative stem anteversion varying from 2.3° ± 5.9° to 22.7° ± 11.6° and the correlation coefficient varying from 0.46 to 0.93. Those methods were majorly based on the complicated femoral coordinate system or 3D CT reconstruction models, and the manual measurement was also inefficient. Furthermore, some studies found that it was difficult to predict postoperative stem anteversion.^7^, ^15^

We speculated that one main reason for the controversy may be the conventional prediction methods for postoperative stem anteversion that are currently used and based on the native femoral anteversion measurement at the location of the femoral head and/or neck.^7^, ^8^, ^9^, ^10^, ^11^ Theoretically, the anteversion of a cementless straight tapered femoral stem should depend on the anteversion of the anatomical locations that fix the stem in the proximal femur, rather than the femoral head and/or neck, which play only a part or no role in fixing the stem. Therefore, prediction of postoperative stem anteversion using conventional native femoral anteversion may not be appropriate.

**Figure 1:**
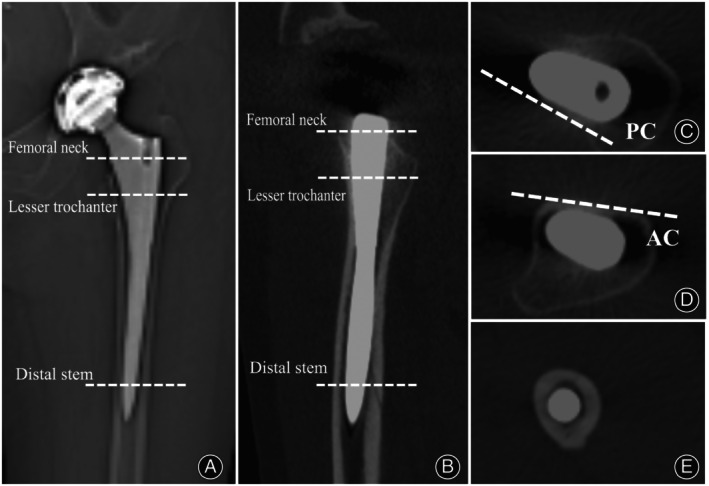
The fixation of the femoral stem in the sagittal plane: (A) The anterior to posterior image after total hip arthroplasty (THA); (B) Computed tomography (CT) reconstruction image in the sagittal plane after THA; (C) Posterior cortex (PC) at the femoral neck; (D) Anterior cortex (AC) at the lesser trochanter (LT); (E) The stem is circular or elliptical at the distal stem.

Although the proximal femoral canal is complicated, the successful fixation of a cementless straight tapered stem depends on stabilization in three dimensions. In the sagittal plane, the femur displays a slight “S” shape, while the stem is straight. Hence, based on the principle of three‐point fixation,^16^, ^17^ the posterior cortex at the femoral neck, the anterior cortex at the lesser trochanter, and the cortex around the distal stem are the three key locations that fix the femoral stem in the sagittal plane (Figure 1). Since the distal stem is circular or elliptical, the anterior cortex at the lesser trochanter and the posterior cortex at the femoral neck are the main anatomical structures that control rotation stability and anteversion of the stem. Hence, whether the anteversion of the anterior cortex at the lesser trochanter level and that of the posterior cortex at the femoral neck level could help predict postoperative stem anteversion remain unknown.

Therefore, this study aimed to (i) explore the predictive role of a new method for postoperative stem anteversion in DDH patients, which was based on the principle of sagittal three‐point fixation and (ii) further analyze the reliability and feasibility of this novel method.

## Methods

### Study Design and Patient Selection

This retrospective study was approved by the ethic committee of the Medical Ethics Committee of Shanghai Ninth People's Hospital, Shanghai Jiao Tong University School of Medicine (SH9H2021‐T238‐2) and the need for informed consent was waived, since the human data that were collected were anonymized. A total of 344 consecutive THAs performed for DDH patients between January 2017 and December 2018 were included. The inclusion criteria were as fellow: (1) patients aged between 25 and 80 years; (2) patients diagnosed with DDH by a senior doctor; (3) the primary THA surgeries were performed by the single surgeon, with the posterolateral approach and “acetabular first” technique with the concept of combined anteversion. The procedure has been described in detail previously.^4^, ^18^ Routine intraoperative fluoroscopy was used to verify the size and position of the final femoral broach to achieve “best‐fit”.

**Figure 2:**
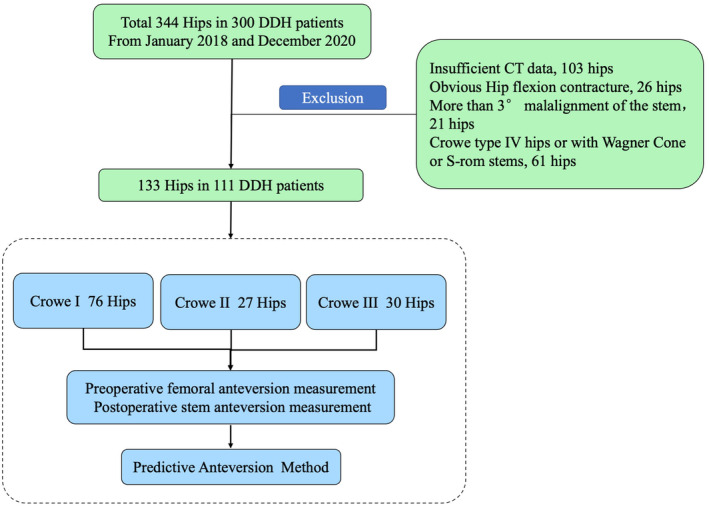
Flowchart depicting data selection and categorization of the study.

In total, 211 hips were excluded from this study because of insufficient preoperative or postoperative CT data (103 hips), obvious flexion contracture of the hip (26 hips), Crowe type IV hips and hips with Wagner Cone or S‐rom stems (47 hips), and more than 3° malalignment of the stem in the coronal and/or sagittal plane (21 hips). The remaining 133 hips (111 patients) were included in the study, including 87 women and 24 men with a mean age of 59.77 ± 11.22 (range, 29–85) years. There were 76 Crowe type I hips, 27 type II hips, and 30 type III hips (Table 1). The single‐wedge stem was used in 49 hips (Accolade, Stryker, Howmedica, Mahwah, NJ, USA), and the double‐wedge stem was used in 84 hips (Figure 2) (Secur‐fit No. 47, Stryker, Howmedica, Mahwah, NJ, USA; Summit No. 37, DePuy, Warsaw, IN, USA).

**TABLE 1:** Patient information.

No. of patients (hips)	111 (133)
Mean age (yrs) (mean ± SD)	59.77 ± 11.22
Gender (male/female)	24/87
BMI (kg/m^2^) (mean ± SD)	24.54 ± 6.12
Crowe type (I/II/III)	76/27/30
Neck height (mm)	16.33 ± 3.59
Stem anteversion (°)	25.33 ± 13.12

Abbreviations: BMI, body mass index; SD, standard deviation.

### CT Data Acquisition and Measurement

CT evaluation was performed as we previously reported^19^ by using the Hitachi Radix Turbo (Tokyo, Japan) (120 kVp, 200 mA, 5‐mm collimation, 5 mm/s table speed, and 5‐mm resolution index) device. By using axial sections passing from the anterior superior iliac spine to the tibial tubercle, patients underwent 1‐mm‐interval CT in the supine position with the hips and knees fully extended and the lower limbs as horizontal and parallel as possible. Pre‐ and postoperative CT data was stored in Digital Imaging and Communication in Medicine (DICOM) format. Femoral measurement was performed using the Radiant DICOM Viewer (version 4.6.9, 64‐bit, Medixant Company, Poland).

The femoral neck height was measured as the vertical distance between the proximal base of the lesser trochanter and the medial edge of the femoral head–neck junction. The femoral neck osteotomy height was measured as the vertical distance between the proximal base of the lesser trochanter and the medial edge of the femoral neck osteotomy plane.

**Figure 3:**
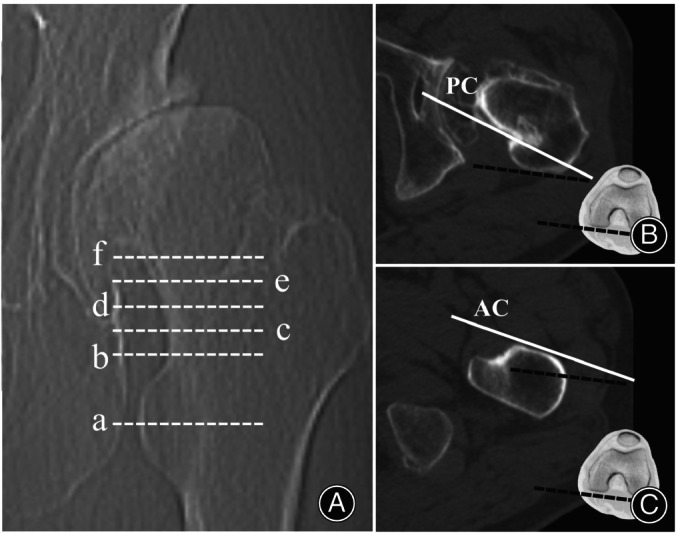
The anteversion measurement of the anterior cortex and posterior cortex. (A) Six measurement levels of the proximal femur: Level a, center of the lesser trochanter; Level b, proximal base of the lesser trochanter; Level c, 5 mm above level b; Level d, 10 mm above level b; Level e, 15 mm above level b; Level f, the femoral head–neck junction. (B) The anteversion of the posterior cortex (PC) at Levels b, c, d, e, and f is measured as the angle formed by the cortical line (white solid line) and the posterior condylar axis (black dashed line). (C) The anteversion of the anterior cortex (AC) at Levels a and b is measured as the angle formed by the cortical line (white solid line) and the posterior condylar axis (black dashed line).

The anteversion of the anterior cortex at two levels of the lesser trochanter (Levels a and b) and the anteversion of the posterior cortex at five levels of the femoral neck (Levels b, c, d, e, and f) were measured *via* preoperative two‐dimensional CT (Figure 3). The levels were as follows: Level a, center of the lesser trochanter; Level b, proximal base of the lesser trochanter; Level c, 5 mm above Level b; Level d, 10 mm above Level b; Level e, 15 mm above Level b; and Level f, femoral head–neck junction (just below the head). First, the anteversion of every cortex was measured as the angle formed by the cortical line and the posterior condylar axis. The posterior condylar axis was defined as the line drawn along the largest femoral condyle on the CT slice.^10^ Based on the principle of three‐point fixation in the sagittal plane (Figure 1), the predictive anteversion by the new method was then calculated as the average anteversion of the anterior cortex at two levels of the lesser trochanter and posterior cortex at five levels of the femoral neck. Thus, 10 predictive values of anteversion from 10 level combinations (Level ab, Level ac, Level ad, Level ae, Level af, Level bb, Level bc, Level bd, Level be, and Level bf) were obtained.

The native femoral anteversion at the femoral head–neck junction (Level f) was measured by the conventional method of Suh et al.,^10^ defined as the angle between the midcortical line and the posterior condylar axis, which was often used for the prediction of postoperative stem anteversion in previous studies.^11^ Postoperative stem anteversion was measured as the angle formed by the stem neck major axis and the posterior condylar axis.^20^

All CT measurements were performed twice over an interval of more than 2 weeks by two orthopaedic surgeons.

### Statistical Analysis

Data were expressed as the mean ± standard deviation. All statistical analyses were performed using SPSS software for Windows (version 18.0; SPSS, Chicago, IL, USA). The intraclass correlation coefficients of interobserver and intraobserver reliabilities were calculated. Normal distribution was tested before any statistical analysis. The difference and correlation between preoperative predictive anteversion and postoperative stem anteversion were analyzed. A *p* value < 0.05 was considered to represent a significant difference. Correlation (*r*) was characterized as poor (0.00–0.20), fair (0.21–0.40), moderate (0.41–0.60), good (0.61–0.80), or excellent (0.81–1.00).

## Results

The intraclass correlation coefficients for intraobserver and interobserver reliabilities were excellent (0.845–0.933 and 0.822–0.946, respectively). For all 133 hips, the mean postoperative stem anteversion was 25.33° ± 13.12°, the mean preoperative femoral neck height was 16.33 ± 3.59 mm, including 37 hips with neck height <15 mm and 96 hips with neck height ≥15 mm (Table 1). The correlation between preoperative femoral neck height and postoperative stem anteversion was fair (*r* = 0.240).

### Anteversion of the Anterior Cortex and Posterior Cortex

As shown in Table 2, for all hips, the anteversion of the anterior cortex at Level a and Level b was 17.18° ± 12.97° and 16.28° ± 13.24°, respectively. The anteversion of the posterior cortex was reduced gradually from 56.32° ± 16.43° at Level b to 21.63° ± 14.56° at Level f.

**TABLE 2:** Anteversion of anterior and posterior cortex.

Position	Level	No.	Anteversion (°)
Anterior cortex	a	133	17.18 ± 12.97
	b	133	16.28 ± 13.24
Posterior cortex	b	133	56.32 ± 16.43
	c	133	45.90 ± 14.16
	d	133	36.21 ± 12.83
	e	96	26.02 ± 12.97
	f	133	21.63 ± 14.56

### Difference between Predictive Anteversion and Postoperative Stem Anteversion

As shown in Table 3, the predictive anteversion was reduced gradually from Level ab to Level af and from Level bb to Level bf with varied differences and correlations between predictive anteversion and postoperative stem anteversion. The predictive anteversion at Level bd (26.24° ± 12.23°) showed no difference with postoperative stem anteversion (25.33° ± 13.12°, *p* = 0.163), with a small difference (0.92° ± 7.52°, −17.00–15.00°) and an excellent correlation (*r* = 0.826). However, the native femoral anteversion at Level f (17.83° ± 13.99°) was significantly less than postoperative stem anteversion, with a difference of −7.49° ± 8.83° and a correlation of 0.790. In general, these data indicated that the predictive anteversion at Level bd could be used as a better predictor of postoperative stem anteversion than native femoral anteversion at Level f.

**TABLE 3:** Difference between predictive/native femoral anteversion and postoperative stem anteversion.

Level	No.	PA/NFA (°)	Stem anteversion (°)	Difference (°)	*p*	*r*
ab	133	36.75 ± 13.66	25.33 ± 13.12	11.42 ± 8.22	<0.001	0.834
ac	133	31.54 ± 12.57	25.33 ± 13.12	6.21 ± 7.72	<0.001	0.820
ad	133	26.70 ± 11.88	25.33 ± 13.12	1.37 ± 7.54	0.038	0.823
ae	96	20.67 ± 11.82	24.12 ± 13.16	−3.46 ± 7.84	<0.001	0.808
af	133	19.41 ± 12.97	25.33 ± 13.12	−5.92 ± 7.76	<0.001	0.823
bb	133	36.30 ± 13.69	25.33 ± 13.12	10.97 ± 7.74	<0.001	0.834
bc	133	31.09 ± 12.80	25.33 ± 13.12	5.76 ± 7.53	<0.001	0.832
bd	133	26.24 ± 12.23	25.33 ± 13.12	0.92 ± 7.52	0.163	0.826
be	96	20.42 ± 12.07	24.28 ± 13.18	−3.70 ± 7.73	<0.001	0.816
bf	133	18.96 ± 13.28	25.33 ± 13.12	−6.37 ± 7.72	<0.001	0.829
f	133	17.83 ± 13.99	25.33 ± 13.12	−7.49 ± 8.83	<0.001	0.790

*Note*: Differences (°) = PA/NFA−stem anteversion.

Abbreviations: NFA, native femoral anteversion; *p*, comparison between PA/NFA with stem anteversion; PA, predictive anteversion; *r*, correlation of PA/NFA with stem anteversion.

### Prediction for Hips with Different Femoral Neck Heights

As shown in Table S1, excellent correlations were shown between predictive anteversions and postoperative stem anteversion for both hips with femoral neck height ≥15 mm and hips with femoral neck height <15 mm.

For hips with femoral neck height ≥15 mm, the predictive anteversion at Level bd showed no statistical difference with postoperative stem anteversion, with a difference of 1.47° ± 7.41°. The native femoral anteversion at Level f was significantly less than the postoperative stem anteversion, with a difference of −9.38° ± 8.22°.

For hips with femoral neck height <15 mm, the predictive anteversion at Levels ad, af, and bd, and native femoral anteversion at Level f showed no statistical difference with postoperative stem anteversion, with a difference of 0.46° ± 7.36°, −2.47° ± 7.46°, −0.52° ± 7.71°, and − 2.59° ± 8.58°, respectively. However, the differences at Levels ad and bd were significantly less than that of Level f (*p* = 0.005 and *p* = 0.029, respectively).

Hence, for hips with different femoral neck heights, the predictive anteversion at Level bd was closer to postoperative stem anteversion than native femoral anteversion at Level f.

### Anteversion Prediction for Hips with Different Crowe Types

For all three Crowe types (Table S2), good to excellent correlations were shown between postoperative stem anteversion and predictive anteversion or native femoral anteversion. The predictive anteversions at Levels ad and bd for type I, Levels ae and bd for type II, and Levels ad, ae, bd, and be for type III showed no statistical differences with postoperative stem anteversions. For all three types, the predictive anteversions at Level bd showed no statistical differences with postoperative stem anteversions, with a difference of 0.49° ± 7.66°, 2.76° ± 7.02°, and 0.33° ± 7.59°, respectively. However, the native femoral anteversion at Level f was significantly less than postoperative stem anteversions for all three types, with a difference of −9.46° ± 8.07°, −5.24° ± 9.15°, and − 4.55° ± 9.35°, respectively.

These data indicated that for hips with different Crowe types, the predictive anteversions at Level bd were closer to postoperative stem anteversion than native femoral anteversion at Level f.

### Anteversion Prediction for Hips with Different Stem Types

Correlations between postoperative stem anteversions and predictive anteversion or native femoral anteversion were good for single‐wedge stems (*r* = 0.688–0.773) and excellent for double‐wedge stems (*r* = 0.813–0.875) (Table S3). The predictive anteversion at Level ad and Level bd showed no statistical difference with postoperative stem anteversion, with a difference of 1.67° ± 7.53° and 1.80° ± 7.37°, respectively, for single‐wedge stems, and 1.19° ± 7.58° and 0.40° ± 7.60°, respectively, for double‐wedge stems. However, the native femoral anteversion at Level f was significantly different from postoperative stem anteversion both for single‐wedge and double‐wedge stems, with a difference of −7.75° ± 8.84° and − 7.35° ± 8.88°, respectively.

Hence, for hips with different stem types, the predictive anteversions at Level ad and Level bd were closer to postoperative stem anteversion compared to native femoral anteversion at Level f.

### Anteversion Prediction for Hips with Different Femoral Anteversion

According to the native femoral anteversion at Level f measured by the conventional method of Suh et al.,^10^ all 133 hips were classified into three groups (Table S4).

For hips with femoral anteversion <0°, the predictive anteversion at Levels ad and bd showed no statistical difference with postoperative stem anteversion, with good correlations, but the native femoral anteversion at Level f showed no correlation with postoperative stem anteversion (*r* = 0.122, *p* = 0.705).

For hips with femoral anteversion 0°–30°, the predictive anteversion at Levels ad and bd showed no statistical difference with postoperative stem anteversion, with good correlations, but the native femoral anteversion at Level f was significantly less than the postoperative stem anteversion (difference = −8.27° ± 8.08°), although with a good correlation.

For hips with femoral anteversion >30°, the predictive anteversion at Levels ad, ae, bd, be, bf, and the native femoral anteversion at Level f showed no statistical difference with postoperative stem anteversion, but good correlations for Levels ad, ae, bd, be, and bf (*r* = 0.629–0.712), and only moderate correlations for Level f (*r* = 0.424).

Hence, for hips with different native femoral anteversion, the predictive anteversions at Level ad and Level bd could be used to predict postoperative stem anteversion.

## Discussion

**Figure 4:**
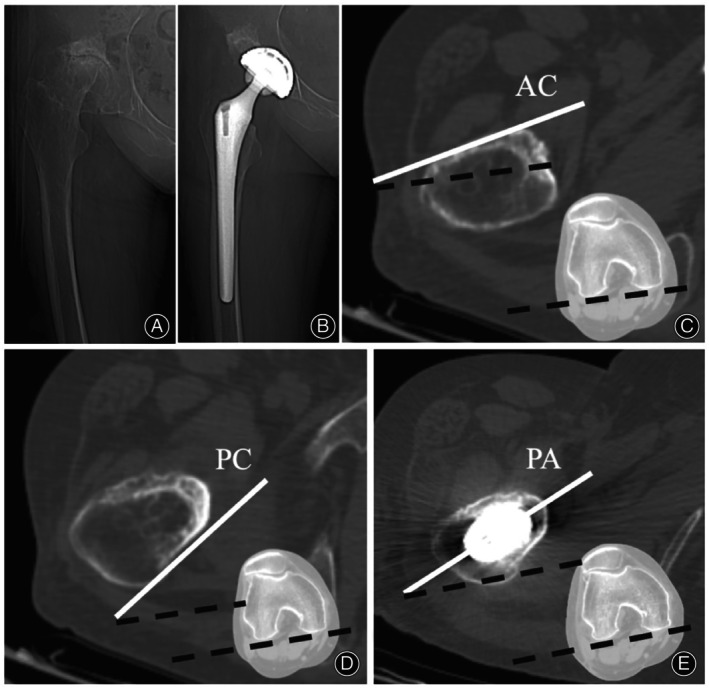
A representative case of anteversion measurement based on our novel method. (A) The preoperative X‐ray of a 66‐year‐old female with a DDH history. (B) The postoperative X‐ray of that patient. (C) The anteversion of the anterior cortex at level b was measured, and the anteversion angle was 12.3°. (D) The anteversion of the posterior cortex at level d was measured, and the anteversion angle was 32.5°. (E) The anteversion of the postoperative prosthesis axis was measured, and the anteversion angle was 22.2°. Based on our method, the predicting postoperative stem anteversion was 22.4°, which was pretty close to the actual stem anteversion (AC, anterior cortex; PC, posterior cortex; PA, prosthesis axis).

In this study, we analyzed the CT data of 133 hips in 111 patients, and proposed a novel and reliable method to predict the postoperative stem anteversion based on the principle of sagittal three‐point fixation. The predictive anteversion method was measured as the average anteversion of the anterior cortex at the lesser trochanter proximal base and posterior cortex 10 mm above the lesser trochanter proximal base (Figure 4).

### Establish a Novel Method for Predicting Postoperative Stem Anteversion

The present study found a poor correlation between the femoral neck osteotomy height and postoperative stem anteversion for Crowe type I‐III DDH hips. Similar to our results, Worlicek et al.^15^, ^21^ reported that the femoral neck osteotomy height did not correlate with postoperative stem anteversion, varus/valgus alignment, or tilt, for non‐DDH hips. The study by Rozis et al.^22^ found that if the classical femoral neck osteotomy technique were adopted, a posterior entry point and penetration of the posterior cortex of the femoral neck would be required to achieve a balanced alignment of the straight stem. Therefore, if a neutral stem is achieved in the coronal and sagittal planes, the height of the femoral neck osteotomy will have no significant effect on the anteversion of the straight stem.

Preoperative estimation of femoral anteversion to predict postoperative stem anteversion helps in the selection of a suitable femoral prosthesis and optimizes the combined anteversion in the “acetabular first” technique in THA for DDH patients.^2^, ^4^ Conventionally, femoral anteversion is measured at the level of the femoral head–neck junction.^9^ However, a variety of differences and correlations between femoral anteversion and postoperative stem anteversion have been reported in previous studies.^11^, ^13^ Similarly, this study demonstrated that for DDH hips with different neck heights, different Crowe types, different stem types, or different femoral anteversions, the differences and correlations measured at Level f (femoral head–neck junction) were also widely varied, with differences ranging from −1.27° ± 8.33° to −13.67° ± 9.47° and correlations ranging from 0.122 (*p* = 0.705, no correlation) to 0.813. Hence, this study further demonstrated the unreliability of conventional prediction methods for postoperative stem anteversion.

### The Reliability and Feasibility of this Novel Method

The femur displays a slight “S” shape in the sagittal plane, and the anterior cortex at the lesser trochanter and the posterior cortex of the femoral neck contribute to the sagittal stability of the straight femoral stem. Our results showed that compared with native femoral anteversion at Level f using the conventional method,^10^, ^11^ the difference between predictive anteversion at Level bd and postoperative stem anteversion was less varied (from 0.33° ± 7.59° to 2.76° ± 7.02°) for different DDH hips, with good to excellent correlations of 0.672–0.858. This indicated that the predictive anteversion at Level bd (average anteversion of the anterior cortex at the lesser trochanter proximal base and posterior cortex 10 mm above the lesser trochanter proximal base) showed a more definite role. Thus, our new predictive method based on the principle of three‐point fixation can predict postoperative stem anteversion more reliably than the native femoral anteversion using the conventional method.

Consistent with the results of a previous study by Taniguchi et al.,^11^ the present results showed that the anteversion of the double‐wedge stem had a better correlation with predictive anteversion than that of the single‐wedge stem. It is believed that the difference is related to the stem design. Double‐wedge stems have cortical contact with the proximal femur in both coronal and sagittal planes, while single‐wedge stems are relatively thin and designed to acquire cortical fixation in the coronal plane.^23^ The single‐wedge stem has certain freedom in the horizontal plane.^17^ However, the native femoral anteversion or predictive anteversion measured by different methods could be different even for the same hip,^9^, ^13^ and every hip is unique. There may be one prediction method that is more suitable for a specific stem or a specific hip than other methods.

### Limitations and Strengths

Based on our study, we invent a novel and reliable method to predict the postoperative stem anteversion, which could be further applied in clinical practice. However, there were limitations to this study. Although there are several methods to measure femoral anteversion and to predict postoperative stem anteversion, our new prediction method was only compared to one of the commonly used methods by Suh et al. on CT slices.^10^, ^11^ In addition, this was a retrospective study in a single center, and many DDH hips were excluded due to incomplete CT data, which may have caused data bias. Thus, a prospective randomized controlled trial with larger sample sizes and higher quality will be further warranted to confirm the results, and then our novel method can be widely used in clinical settings.

## Conclusion

This study demonstrated that for DDH hips with different neck heights, different Crowe types, different stem types, or different femoral anteversions, the native femoral anteversion measured at the femoral head–neck junction showed widely varied differences and correlations with postoperative stem anteversion. Compared with native femoral anteversion, the predictive anteversion measured as the average anteversion of anterior cortex at the lesser trochanter proximal base and posterior cortex 10 mm above the lesser trochanter proximal base showed less varied differences and correlations with postoperative stem anteversion. Therefore, our new predictive method based on the principle of sagittal three‐point fixation was more reliable for prediction of postoperative stem anteversion.

## Author Contributions

All authors had full access to the data in the study and take responsibility for the integrity of the data and the accuracy of the data analysis. Conceptualization: YH.H., ZY.S., and ZJ.Z. Methodology: YH.H. Software: ZY.S. Validation: JW.Z., DG.Y., and YH.H. Formal analysis: ZY.S. Investigation: JW.Z. Resources: ZY.S. Data curation: DG.Y. Writing—original draft preparation: YH.H., ZY.S. Writing—review and editing: ZJ.Z., DG.Y., HW.L. Visualization: HW.L. Supervision: MN.Y. Project administration: ZJ.Z. Funding acquisition: DG.Y., and ZJ.Z. All authors have read and agreed to the published version of the manuscript. All authors have read and approved the final submitted manuscript.

## Funding Information

This work was supported by grants from the National Natural Science Foundation of China (No. 81772361), Shanghai Clinical Medical Center (Grant Number 2017ZZ01023), and Shanghai Municipal Key Clinical Specialty.

## Conflict of Interest Statement

The authors declare that they have no competing interests.

## Ethics Statement

This study was conducted according to the guidelines of the Declaration of Helsinki, and approved by the Institutional Review Board of Shanghai Ninth People's Hospital, Shanghai Jiaotong University School of Medicine. Meanwhile, the ethics committee of Shanghai Ninth People's Hospital waived the need for informed consent (protocol code SH9H2021‐T238‐2).

## Supporting information


**Table S1.** Anteversion prediction for hips with different femoral neck height.

**Table S2.** Anteversion prediction for hips with different Crowe types.

**Table S3.** Anteversion prediction for hips with different stem types.

**Table S4.** Anteversion prediction for hips with different native femoral anteversion.
