# Supplementary material for: A New Method to Predict Postoperative Stem Anteversion in Total Hip Arthroplasty for Developmental Dysplasia of the Hip
Source: Orthop Surg. 2024 Mar 20;16(5):1101–8. doi: 10.1111/os.14037 (PMC11062849; doi:10.1111/os.14037)
Supplement: Supplementary file 1 — Table S1. Anteversion prediction for hips with different femoral neck height. [file OS-16-1101-s001.docx]

| **Supplement Table 1. Anteversion prediction for hips with different femoral neck height** | | | | | | | | | |
| --- | --- | --- | --- | --- | --- | --- | --- | --- | --- |
| Level | Femoral neck height ≥15mm (96hips) | | | |  | Femoral neck height <15mm (37hips) | | | |
|  | PA/NFA (°) | Difference (°) | *P* | r |  | PA/NFA (°) | Difference (°) | *P* | r |
| ab | 34.37±13.22 | 10.25±8.28 | <0.001 | 0.803 |  | 42.92±12.98 | 14.46±7.31 | <0.001 | 0.838 |
| ac | 30.03±12.77 | 5.91±8.14 | <0.001 | 0.803 |  | 35.46±11.29 | 7.00±6.53 | <0.001 | 0.857 |
| ad | 25.84±11.88 | 1.72±7.62 | 0.030 | 0.820 |  | 28.92±11.76 | 0.46±7.36 | 0.706 | 0.821 |
| ae | 20.67±11.82 | -3.46±7.84 | <0.001 | 0.808 |  | / | / | / | / |
| af | 16.87±12.31 | -7.25±7.49 | <0.001 | 0.829 |  | 25.99±12.46 | -2.47±7.46 | 0.052 | 0.824 |
| bb | 34.12±13.22 | 10.00±7.81 | <0.001 | 0.825 |  | 41.94±13.45 | 13.48±7.04 | <0.001 | 0.856 |
| bc | 29.78±12.91 | 5.66±7.90 | <0.001 | 0.817 |  | 34.48±12.01 | 6.02±6.57 | <0.001 | 0.859 |
| bd | 25.59±12.07 | 1.47±7.41 | 0.055 | 0.831 |  | 27.94±12.64 | -0.52±7.71 | 0.683 | 0.814 |
| be | 20.42±12.07 | -3.70±7.73 | <0.001 | 0.816 |  | / | / | / | / |
| bf | 16.62±12.59 | -7.50±7.44 | <0.001 | 0.834 |  | 25.01±13.28 | -3.45±7.77 | 0.010 | 0.822 |
| f | 14.74±12.73 | -9.38±8.22 | <0.001 | 0.799 |  | 25.87±14.07 | -2.59±8.58 | 0.074 | 0.799 |
| Differences (°) = PA/NFA - stem anteversion;  PA, predictive anteversion; NFA, native femoral anteversion; *P*, comparison between PA/NFA with stem anteversion; r, correlation of PA/NFA with stem anteversion. | | | | | | | | | |
