# Supplementary material for: A New Method to Predict Postoperative Stem Anteversion in Total Hip Arthroplasty for Developmental Dysplasia of the Hip
Source: Orthop Surg. 2024 Mar 20;16(5):1101–8. doi: 10.1111/os.14037 (PMC11062849; doi:10.1111/os.14037)
Supplement: Supplementary file 2 — Table S2. Anteversion prediction for hips with different Crowe types. [file OS-16-1101-s003.docx]

| **Supplement Table 2. Anteversion prediction for hips with different Crowe types** | | | | | | | | | | | | | | |
| --- | --- | --- | --- | --- | --- | --- | --- | --- | --- | --- | --- | --- | --- | --- |
|  | Crowe type I (76hips) | | | |  | Crowe type II (27hips) | | | |  | Crowe type III (30hips) | | | |
| Level | PA/NFA (°) | Difference (°) | *P* | r |  | PA/NFA (°) | Difference (°) | *P* | r |  | PA/NFA (°) | Difference (°) | *P* | r |
| ab | 33.00±12.95 | 9.31±8.54 | <0.001 | 0.788 |  | 38.35±12.83 | 12.95±5.94 | <0.001 | 0.893 |  | 44.81±12.68 | 15.39±7.54 | <0.001 | 0.819 |
| ac | 28.55±12.63 | 4.86±8.40 | <0.001 | 0.791 |  | 34.13±12.41 | 8.73±6.64 | <0.001 | 0.862 |  | 36.80±10.50 | 7.38±6.09 | <0.001 | 0.872 |
| ad | 24.35±11.81 | 0.66±7.90 | 0.469 | 0.808 |  | 28.72±12.45 | 3.32±6.88 | 0.019 | 0.852 |  | 30.82±10.35 | 1.40±7.07 | 0.287 | 0.822 |
| ae | 19.53±11.85 | -4.52±8.04 | <0.001 | 0.815 |  | 23.13±11.62 | -1.29±7.21 | 0.470 | 0.806 |  | 23.53±11.94 | -0.58±6.67 | 0.906 | 0.741 |
| af | 16.14±11.96 | -7.55±7.52 | <0.001 | 0.828 |  | 21.58±13.00 | -3.82±7.69 | 0.016 | 0.823 |  | 25.73±13.06 | -3.69±7.65 | 0.013 | 0.821 |
| bb | 32.83±12.95 | 9.14±7.99 | <0.001 | 0.815 |  | 37.79±13.54 | 12.93±5.60 | <0.001 | 0.911 |  | 43.74±12.84 | 14.31±7.56 | <0.001 | 0.851 |
| bc | 28.38±12.85 | 4.69±8.20 | <0.001 | 0.804 |  | 33.58±13.22 | 8.18±6.48 | <0.001 | 0.877 |  | 35.73±10.71 | 6.30±6.15 | <0.001 | 0.869 |
| bd | 24.18±12.03 | 0.49±7.66 | 0.579 | 0.822 |  | 28.16±13.40 | 2.76±7.02 | 0.051 | 0.858 |  | 29.75±10.89 | 0.33±7.59 | 0.815 | 0.796 |
| be | 19.30±12.12 | -4.75±7.78 | <0.001 | 0.829 |  | 23.13±11.80 | -1.29±6.91 | 0.009 | 0.824 |  | 22.86±12.20 | -1.25±7.81 | 0.590 | 0.797 |
| bf | 15.97±12.27 | -7.72±7.41 | <0.001 | 0.835 |  | 21.03±14.05 | -4.37±8.06 | 0.012 | 0.860 |  | 24.66±13.26 | -4.77±7.75 | 0.002 | 0.820 |
| f | 14.23±12.23 | -9.46±8.07 | <0.001 | 0.803 |  | 20.16±15.10 | -5.24±9.15 | 0.006 | 0.807 |  | 24.88±14.46 | -4.55±9.35 | 0.013 | 0.768 |
| Differences (°) = PA/NFA - stem anteversion;  PA, predictive anteversion; NFA, native femoral anteversion; *P*, comparison between PA/NFA with stem anteversion; r, correlation of PA/NFA with stem anteversion. | | | | | | | | | | | | | | |
