# Supplementary material for: A New Method to Predict Postoperative Stem Anteversion in Total Hip Arthroplasty for Developmental Dysplasia of the Hip
Source: Orthop Surg. 2024 Mar 20;16(5):1101–8. doi: 10.1111/os.14037 (PMC11062849; doi:10.1111/os.14037)
Supplement: Supplementary file 3 — Table S3. Anteversion prediction for hips with different stem types. [file OS-16-1101-s002.docx]

| **Supplement Table 3. Anteversion prediction for hips with different stem types** | | | | | | | | | |
| --- | --- | --- | --- | --- | --- | --- | --- | --- | --- |
| Level | Single-wedge Stem (49hips) | | | |  | Double-wedge Stem (84hips) | | | |
|  | PA/NFA (°) | Difference (°) | *P* | r |  | PA/NFA (°) | Difference (°) | *P* | r |
| ab | 34.15±10.42 | 10.05±8.48 | <0.001 | 0.688 |  | 38.27±15.08 | 12.22±8.01 | <0.001 | 0.852 |
| ac | 29.14±11.35 | 5.04±8.43 | <0.001 | 0.715 |  | 32.94±13.09 | 6.90±7.23 | <0.001 | 0.863 |
| ad | 25.77±10.80 | 1.67±7.53 | 0.126 | 0.761 |  | 27.23±12.50 | 1.19±7.58 | 0.155 | 0.843 |
| ae | 19.74±9.53 | -3.82±7.72 | 0.004 | 0.720 |  | 21.27±13.15 | -3.21±7.98 | 0.003 | 0.839 |
| af | 17.65±11.59 | -6.45±7.76 | <0.001 | 0.765 |  | 20.43±13.68 | -5.62±7.79 | <0.001 | 0.845 |
| bb | 34.28±10.41 | 10.18±8.24 | <0.001 | 0.705 |  | 37.48±15.22 | 11.43±7.44 | <0.001 | 0.874 |
| bc | 29.27±11.55 | 5.17±8.48 | <0.001 | 0.703 |  | 32.15±13.43 | 6.11±6.95 | <0.001 | 0.875 |
| bd | 25.90±10.87 | 1.80±7.37 | 0.093 | 0.773 |  | 26.44±13.02 | 0.40±7.60 | 0.634 | 0.848 |
| be | 19.73±9.64 | -3.84±7.63 | 0.004 | 0.728 |  | 20.87±13.49 | -3.61±7.85 | 0.001 | 0.846 |
| bf | 17.78±11.72 | -6.32±7.71 | <0.001 | 0.772 |  | 19.64±14.14 | -6.41±7.77 | <0.001 | 0.850 |
| f | 16.35±12.59 | -7.75±8.84 | <0.001 | 0.727 |  | 18.70±14.75 | -7.35±8.88 | <0.001 | 0.813 |
| Differences (°) = PA/NFA - stem anteversion;  PA, predictive anteversion; NFA, native femoral anteversion; *P*, comparison between PA/NFA with stem anteversion; r, correlation of PA/NFA with stem anteversion. | | | | | | | | | |
