# Supplementary material for: A New Method to Predict Postoperative Stem Anteversion in Total Hip Arthroplasty for Developmental Dysplasia of the Hip
Source: Orthop Surg. 2024 Mar 20;16(5):1101–8. doi: 10.1111/os.14037 (PMC11062849; doi:10.1111/os.14037)
Supplement: Supplementary file 4 — Table S4. Anteversion prediction for hips with different native femoral anteversion. [file OS-16-1101-s004.docx]

| **Supplement Table 4. Anteversion prediction for hips with different native femoral anteversion** | | | | | | | | | | | | | | |
| --- | --- | --- | --- | --- | --- | --- | --- | --- | --- | --- | --- | --- | --- | --- |
|  | Femoral Anteversion<0° (12hips) | | | |  | Femoral Anteversion 0°-30° (97hips) | | | |  | Femoral Anteversion >30° (24hips) | | | |
| Level | PA/NFA (°) | Difference (°) | *P* | r |  | PA/NFA (°) | Difference (°) | *P* | r |  | PA/NFA (°) | Difference (°) | *P* | r |
| ab | 16.23±10.56 | 7.55±8.25 | 0.009 | 0.661 |  | 34.94±9.88 | 11.40±8.50 | <0.001 | 0.660 |  | 54.31±7.37 | 13.44±6.45 | <0.001 | 0.644 |
| ac | 11.65±10.14 | 2.96±10.42 | 0.346 | 0.426 |  | 30.03±8.82 | 6.49±7.76 | <0.001 | 0.699) |  | 47.59±6.91 | 6.73±5.73 | <0.001 | 0.707 |
| ad | 9.54±8.75 | 0.85±6.94 | 0.679 | 0.704 |  | 24.89±8.13 | 1.34±7.94 | 0.099 | 0.674 |  | 42.59±8.16 | 1.72±6.29 | 0.193 | 0.693 |
| ae | 2.96±9.41 | -6.32±6.64 | 0.015 | 0.769 |  | 20.23±8.08 | -3.13±8.10 | 0.001 | 0.666 |  | 39.73±7.12 | -3.07±7.12 | 0.184 | 0.651 |
| af | -0.53±7.12 | -9.22±8.36 | 0.003 | 0.504 |  | 17.17±8.22 | -6.37±7.66 | <0.001 | 0.700 |  | 38.40±7.39 | -2.46±6.96 | 0.096 | 0.585 |
| bb | 13.90±9.07 | 5.21±7.27 | 0.030 | 0.685 |  | 34.53±9.19 | 10.99±7.75 | <0.001 | 0.705 |  | 54.62±7.71 | 13.75±6.50 | <0.001 | 0.652 |
| bc | 9.32±8.43 | 0.63±9.53 | 0.823 | 0.422 |  | 29.62±8.51 | 6.08±7.47 | <0.001 | 0.718 |  | 47.91±7.23 | 7.04±5.75 | <0.001 | 0.714 |
| bd | 7.20±7.26 | -1.48±6.19 | 0.424 | 0.744 |  | 24.48±8.08 | 0.93±7.95 | 0.250 | 0.672 |  | 42.90±8.34 | 2.04±6.17 | 0.120 | 0.712 |
| be | 1.51±8.07 | -7.76±6.80 | 0.006 | 0.738 |  | 20.02±8.08 | -3.34±7.92 | <0.001 | 0.682 |  | 40.32±6.90 | -2.48±6.51 | 0.235 | 0.712 |
| bf | -2.86±6.51 | -11.55±8.70 | 0.001 | 0.434 |  | 16.77±7.93 | -6.78±7.41 | <0.001 | 0.720 |  | 38.72±7.33 | -2.15±6.57 | 0.123 | 0.629 |
| f | -4.98±3.45 | -13.67±9.47 | <0.001 | 0.122 |  | 15.27±7.98 | -8.27±8.08 | <0.001 | 0.659 |  | 39.60±7.64 | -1.27±8.33 | 0.463 | 0.424 |
| Differences (°) = PA/NFA - stem anteversion;  PA, predictive anteversion; NFA, native femoral anteversion; *P*,comparison between PA/NFA with stem anteversion; r, correlation of PA/NFA with stem anteversion. | | | | | | | | | | | | | | |
